# Preclinical investigations and a first-in-human phase 1a trial of JS007, a novel anti-CTLA-4 antibody, in patients with advanced solid tumors

**DOI:** 10.1186/s40164-024-00567-7

**Published:** 2024-10-01

**Authors:** Chenfei Zhou, Jinling Jiang, Xiaojun Xiang, Hongli Liu, Guowu Wu, Ruichao Zeng, Tong Lu, Mengqi Zhang, Yuteng Shen, Min Hong, Jun Zhang

**Affiliations:** 1 Department of Oncology, Ruijin Hospital, Shanghai Jiao Tong University School of Medicine, Shanghai, China; 2 Department of Oncology, The First Affiliated Hospital, Jiangxi Medical College, Nanchang University, Nanchang, China; 3 Cancer Center, Union Hospital, Tongji Medical College, Huazhong University of Science and Technology, Wuhan, China; 4 Department of Medical Oncology, Cancer Center, Meizhou People’s Hospital (Huangtang Hospital), Meizhou, China; 5 Shanghai Junshi Biosciences Co., Ltd, Shanghai, China

**Keywords:** CTLA-4, JS007, Monoclonal antibody, Solid tumors, Immunotherapy

## Abstract

**Background:**

Blocking cytotoxic T lymphocyte-associated antigen-4 (CTLA-4) shows substantial antitumor efficacy. Here, we report the preclinical data and outcomes of a first-in-human phase 1a trial of JS007, a novel anti-CTLA-4 antibody, in advanced solid tumors.

**Methods:**

In preclinical studies, both in vitro characteristics and in vivo characteristics of JS007 were investigated. The clinical trial included a dose escalation phase and a dose expansion phase. Eligible patients with previously treated advanced solid tumors were enrolled. In the dose escalation phase, JS007 was administered intravenously every 3 weeks at doses of 0.03, 0.3, 1, 3, and 10 mg/kg. Then, 3 and 10 mg/kg were chosen for the dose expansion phase. The primary endpoints included the maximum tolerated dose (MTD) of JS007 based on dose-limiting toxicities (DLTs) and safety.

**Results:**

JS007 could effectively bind to CTLA-4 and induce an immune response in vitro. Potent in vivo antitumor activity of JS007 was observed. Increased T cell infiltration and T regulatory (Treg) cell depletion in tumor microenvironment of cancer cell xenografts were detected after treated with JS007. Pharmacological analysis in experimental animals showed a dose-proportional increase in exposure. In the clinical trial, a total of 28 patients were treated with JS007 across 5 dose levels. No DLTs occurred. The MTD did not reach at the highest dose tested (10 mg/kg). Twenty-three (82.1%) patients experienced at least one treatment-related adverse event (TRAE). The incidence of Grade ≥ 3 TRAEs was 28.6% (8/28) with alanine aminotransferase increase (7.1%, 2/28) being the most frequently reported TRAE. No severe immune-related adverse event (irAE) occurred. Pharmacological profiles of JS007 in patients were similar to those in animal models. Serum concentration of JS007 showed a dose-dependent escalation, and the half-life of JS007 was 9.4 ~ 12.2 days. Treatment-induced anti-drug antibody was detected in 2 patients. The disease control rate was 50% (14/28), and the median overall survival was 14.7 months.

**Conclusions:**

JS007 preliminarily demonstrates good tolerance and encouraging antitumor activity in patients with previously treated advanced solid tumors.

**Trial registration:**

ClinicalTrials.gov identifier: NCT05049265 (Sep 20, 2021).

**Supplementary Information:**

The online version contains supplementary material available at 10.1186/s40164-024-00567-7.

## Background

In recent decades, immune checkpoint inhibitors (ICIs) have made a breakthrough in the treatment paradigms of cancer [1]. Cytotoxic T lymphocyte-associated antigen-4 (CTLA-4), belongs to the CD28 family receptors, which expresses on T regulatory (Treg) cells and can be upregulated by activated effector T cells as a critical immune checkpoint. The interaction of CTLA-4 and B7 (CD80 or CD86) inhibits signal transmission to T cells, which plays a key role in maintaining homeostasis of immune responses as well as in inducing immune escape of tumor cells. Blocking this inhibitory effect can maintain and activate the antitumor function of effector T cells, which enables CTLA-4 become a potent target of cancer immunotherapy [2, 3].

Ipilimumab, a human anti-CTLA-4 monoclonal antibody (mAb), is the first ICI approved by the US Food and Drug Administration (FDA) as a monotherapy for metastatic melanoma [4, 5]. Currently, ipilimumab combination with nivolumab, a programmed cell death protein 1 (PD-1) inhibitor, has been approved to treat various solid tumors, including melanoma [6], renal cell carcinoma [7], non-small cell lung cancer (NSCLC) [8, 9], metastatic colorectal cancer with microsatellite instability high or mismatch repair deficiency [10], hepatocellular carcinoma (HCC) [11], malignant pleural mesothelioma [12], and esophageal cancer [13]. Tremelimumab is a humanized anti-CTLA-4 IgG2 mAb with preliminary efficacy in metastatic melanoma as monotherapy [14, 15]. However, no positive outcomes were observed in the randomized controlled phase 3 trials [16, 17]. Till now, only two combination regimens of tremelimumab have been approved (combination with durvalumab for HCC [18]; combination with durvalumab plus platinum-based chemotherapy for NSCLC [19]). Several CTLA-4 inhibitors, such as botensilimab [20] and gotistobart [21], are also being investigated, and have shown good tolerance and encouraging antitumor effect.

JS007 is a novel humanized anti-CTLA-4 IgG1 mAb adopting a unique double “wedge-into-hole” binding model which is responsible for its high-affinity binding to CTLA-4. The crystal determination showed that two tyrosine residues of JS007 acted like two wedge anchors and inserted into the cavities of CTLA-4 protein which are formed by BC loop, DE loop, and FG loop. The preclinical study indicated that JS007 could effectively block the CTLA-4/CD80 immune inhibitory signaling pathway, promote T-cell activation and proliferation, thereby boost the immune response to eliminate tumor cells [22].

Here, we reported the preclinical data of JS007 and the outcomes of a phase 1a, first-in-human clinical trial that aimed to assess the safety, pharmacological profiles, and preliminary antitumor activity of JS007 in patients with previously treated advanced solid tumors.

## Methods

### Assessment of CTLA-4 and Fc receptors affinity of JS007

JS007, which was produced as previously described [22], was captured by CM5 chips that were previously coupled with 40 µg/mL of goat anti-human Fc fragment antibody (ImmunoResearch, 109–001–008), followed by binding with different concentrations of His-tagged recombinant human CTLA-4 extracellular domain (ECD). Ipilimumab, which was manufactured in-house according to the published patent, was used as a reference anti-CTLA-4 mAb in preclinical experiments. Anti-KLH IgG1 manufactured in-house was used as a negative control. Consecutive dynamic binding signals were obtained. The affinity was calculated by fitting the data using the kinetic model of Biacore T200 Evaluation Software 3.0 (GE Healthcare Life Sciences).

JS007 belongs to the human IgG1/κ subtype. The affinity of JS007 for Fc receptors was determined using Biacore (GE Healthcare Life Sciences), in which anti-His tag antibody was coupled to a CM5 chip surface, followed by capturing the His-tagged recombinant human FcγRIIIa (CD16a) V176 (Junmeng Biomedical, 20200421), FcγRIIIa (CD16a) F176 (Junmeng Biomedical, 20200421), FcγRIIa (CD32a) R167 (Sino Biological, 10734-H08C), FcγRI (CD64) (Sino Biological, 10256-H08S), and FcRn (Sino Biological, CT009-H08H). The test antibody was then gradient-diluted, and binding signals were detected.

### Direct cytotoxicity test

The direct cytotoxicity of JS007 mediated peripheral blood mononuclear cells (PBMCs, MiaoTong Biotech Company, Donor No. P122030210C) on CHO-hCTLA-4 cells was tested. Serial concentrations of JS007 were incubated with PBMCs and CHO-hCTLA-4 coculture system at a ratio of 25:1 for 4 h. Then, cell supernatants were collected, and the release of lactate dehydrogenase (LDH) in the supernatants was detected using an LDH assay kit (BioVision, K311-400).

### JS007 induced T cell activation test

T cell activation induced by JS007 was detected by Staphylococcal enterotoxin B (SEB) model. SEB was a superantigen that could activate T cells by binding to specific TCR Vβ regions and MHC-II molecules, then inducing the release of various cytokines. PBMCs from different donors (ALLCELLS, LP200520, LP200610, and LP200603) were incubated with JS007, PC-mAb, toripalimab (anti-PD-1 antibody), and hIgG1 control, followed by the addition of SEB (Toxin Technology, BT202red). The supernatant was collected and mixed with P-phycoerythrin (BD CBA human IL2 Flex set, 558270). Then the mixture was loaded and analyzed in the flow cytometer (BD. Canto) to determine the MFI-PE intensity of IL-2 bead, and then the IL-2 concentration was calculated based on the standard curve.

### Antibody-dependent cellular cytotoxicity (ADCC) activation test

An ADCC reporter gene system was constructed to assess JS007-mediated ADCC activity. The Jurkat-ADCC cells as effector cells with overexpression of FcγRIIIa and downstream fluorescent reporter genes, and CHO-hCTLA-4 cells as target cells were both in-house manufactured. Upon receptor engagement, expression of fluorescent reporter genes in activated effector cells was monitored by adding the fluorescent substrate.

### In vivo antitumor effect of JS007 in humanized mice MC38 xenograft model

Dual hPD-1/hCTLA-4 humanized mice were obtained from Biocytogen Co. Ltd (strain: C57BL/6-*Pdcd1*^*tm1(PDCD1)Bcgen*^*Ctla4*^*tm1(CTLA4)Bcgen*^/Bcgen, Animal Production License No. SCXK (Su) 2016-0062). All mice were kept in specific pathogen-free conditions, and the study was conducted in compliance with applicable regulations for care and use of laboratory animals at Immune Technology (Suzhou) Corp. All procedures were approved by the local Institutional Animal Care and Use Committee (IACUC) of the company lab.

Mouse colon cancer MC38 cells (1 × 10^6^) were inoculated subcutaneously into the right posterior back of the humanized mice. When the tumor size reached about 90 mm^3^, the mice were randomly divided into six groups and intraperitoneally injected with JS007 (0.3 mg/kg), ipilimumab (0.3 mg/kg), toripalimab (0.3 mg/kg), JS007 + toripalimab (0.3 mg/kg + 0.3 mg/kg), or ipilimumab + toripalimab (0.3 mg/kg + 0.3 mg/kg) on Day 0, Day 3 and Day 7. Antibody of hIgG1 (0.3 mg/kg) was used as negative control. The tumor growth was monitored twice a week, and the tumor volume (Tv) was calculated by the formula: ½length × width. Tumor growth inhibition (TGI) ratio was calculated by the formula: 1-(Ti-T0)/(Vi-V0) × 100% (Ti: Tv of the treatment group on day i of drug administration; T0: Tv of the treatment group on day 0 of drug administration; Vi: Tv of the NC group on day i; V0: Tv of the NC group on day 0).

At necropsy, tumor tissues of individual animals were harvested and cut into pieces of about 1–3 mm, then dissociated with a tumor tissue dissociation kit (MACS, Cas No. 130-096-730) according to manufacturer’s instruction before being filtered with a 40 μm cell filter. About two million cells were resuspended and transferred into Fluorescence-activated cell sorting tubes, then incubated with APC anti-mouse/Rat Foxp3 (Invitrogen, Cas No. 17-5773-82), FITC anti-mouse CD4 (Biolegend, Cas No. 100406), and PE-CY7 anti-mouse CD3e (BD, Cas No. 552774). A flow cytometry (BD, LSR Fortessa) was applied to analyze the tumor-infiltrated cells (TILs).

### Pharmacokinetics (PK) study of JS007 in non-human primate (NHP) model

Experimental animals (Rhesus monkey) for single-dose pharmacokinetic study and repeat-dose pharmacokinetic study were purchased from Sichuan Hengshu Biotechnology Co. Ltd with Certificate No. SYXK (Shanghai) 2019-0009. All procedures in compliance with all applicable guidelines were approved and monitored by the local IACUC following the protocol of “Pharmacokinetic Study of JS007 Injection after Single Intravenous Infusion in Rhesus Monkeys” (Study No. S19052PK1) and “A 4-week Repeated Dose Toxicity Study of JS007 Injection via Intravenous Injection in Rhesus Monkeys with a 4-week Recovery Period” (Study No. 19052RD01). The single dose PK of JS007 was assessed in experimental animals after administration of a single intravenous infusion at dose levels of 0.3, 1, or 3 mg/kg, respectively. Each dose group consisted of 6 animals (3 males and 3 females). The blood samples were collected on 0.25 h, 0.5 h, 2 h, 4 h, 8 h, 24 h, 48 h, 96 h, 168 h, 240 h, 336 h, 504 h, 672 h, 840 h, and 1008 h following the dose and processed to serum samples after coagulation and centrifugation. The samples were stored at -80 °C before assay.

As a part of the Good Laboratory Practice toxicity study, the repeat-dose pharmacokinetic study (also known as toxicokinetic study) was conducted in compliance with US FDA CFR Part 58, Good Laboratory Practice for non-clinical studies. A standard panel of toxicological and pathological parameters had been evaluated according to ICH S9 “Guidance for Industry S9 Nonclinical Evaluation for Anticancer Pharmaceuticals” and FDA Redbook 2000: IV.C.3b “Short-Term Toxicity Studies with Non-Rodents, 2003”. The execution and interpretation of the study were done by the qualified study director, quality control inspectors, pathologist, and contributed scientist, in order to determine the highest non-severely toxic dose (HNSTD). During the repeated-dose PK analysis, experimental animals were divided into 4 groups with 5 animals per gender in each group and were treated with JS007 intravenously once weekly five times at dose levels of 3, 10, and 30 mg/kg. Blood samples were collected on 0.083 h, 2 h, 6 h, 24 h, 72 h, 120 h, and 168 h following the 1st and 4th dosing. The concentration of JS007 in serum samples of experimental animals was measured by Enzyme-linked Immunosorbent Assay (ELISA) with a validated methodology. The principle of the method involves coating the enzyme plate with HXT hCTLA-4 ECD his (Suzhou Junmeng Biomedical Technology Co. Batch: No. 20181224), adding the test samples, followed by addition of Human IgG-heavy and light chain monkey-adsorbed Antibody Goat Polyclonal Conjugate HRP (BETHYL. Batch: A80-319P-24) as the detection antibody. The PK curve fitting and parameters calculation including maximum observed concentration (C_max_), area under the plasma drug concentration-time curve (AUC), Clearance (CL), and elimination half-life (T_1/2_), were performed by using Phoenix WinNonlin (Certara, Version 8.1).

### Phase 1a study design

This was a single-arm, multicenter, open-label, multiple-dose trial conducted in 4 centers around China. The study consisted of a dose escalation phase and a dose expansion phase. This study was performed in compliance with the Declaration of Helsinki and Good Clinical Practice guidelines. The written informed consents were provided by all patients before enrollment. Study procedures were approved by the independent ethics committee at each participating site.

### Patients

Patients aged 18–75 years, with histologically or cytologically confirmed advanced or recurrent solid tumors and failed with previous standard treatment, or those who had no available standard treatment or refused to receive standard treatment; had at least one measurable lesion according to Response Evaluation Criteria in Solid Tumors (RECIST) version 1.1 [23]; had an Eastern Cooperative Oncology Group performance status (ECOG PS) score of 0 or 1; with a life expectancy of ≥ 3 months; with no prior exposure to anti-CTLA-4 drugs; had adequate organ function were eligible to be enrolled.

Key exclusion criteria included central nervous system metastases; had received immunosuppressive agents within 2 weeks before the first dose of study drug including glucocorticoids at dose equivalent to prednisone > 10 mg/day; systemic anticancer therapy (including radiotherapy, chemotherapy, hormone therapy, surgery, or molecular-targeted therapy) within 4 weeks before study drug was firstly administrated, or unrecovered toxicities from prior anticancer treatment, defined as having not resolved to National Cancer Institute Common Terminology Criteria for Adverse Events (NCI-CTCAE) Grade 1 or below except for alopecia.

### Endpoints

The primary endpoints were the maximum tolerated dose (MTD), the incidence and severity of dose-limiting toxicities (DLTs) events, adverse events (AEs), serious adverse events (SAEs), and immune-related adverse events (irAEs). The secondary endpoints were the PK parameters including C_max_, time to maximum observed concentration, AUC_0 − t_, AUC_0−∞_, T_1/2_, CL, volume of distribution, and anti-drug antibody (ADA). The exploratory endpoints were objective response rate (ORR), duration of response (DoR), disease control rate (DCR), progression-free survival (PFS), and overall survival (OS). ORR was defined as the proportion of patients with a confirmed best overall response of complete response (CR) or partial response (PR). DoR was defined as the time from the initial response (CR or PR, which was confirmed later) to the time of disease progression or death of any cause, whichever came first. DCR was defined as the proportion of patients with the best overall response of CR or PR or stable disease (SD). PFS was defined as the time from the first dose of the study drug to the time of documented radiological disease progression or death of any cause, whichever came first. OS was defined as the time from the first dose of the study drug to the time of death of any cause.

### Procedures

The study was initiated with an accelerated titration scheme in which one patient was enrolled at the 0.03 mg/kg dose level. If no DLT was observed, the study proceeded following a standard 3 + 3 design with sequential enrollment of three patients at three dose levels of 0.3, 1, and 3 mg/kg. The dose level of 10 mg/kg was added per the suggestion of the Safety Monitoring Committee. JS007 was administered intravenously every 3 weeks. DLT was evaluated within 21 days after the first dose of JS007. If no DLT was reported by the first 3 patients in one dose level, the study proceeded to the next dose level. If 1 patient had DLT, 3 more patients would be enrolled at the current dose level. If one or more of the added 3 patients had DLTs, the dose escalation would be terminated, and the previous dose was considered as the MTD. If the additional 3 patients had no DLT, the dose escalation would be forwarded to the next dose level. If ≥ 2 out of 3 patients in a certain dose level had DLTs, the dose escalation would be terminated, or an intermediate dose would be determined by the investigator and sponsor for continued observation. Then this dose was the intolerable dose, and the previous dose was MTD. In the dose expansion phase, two dose levels were selected based on the results of the dose escalation phase, including safety, efficacy, and pharmacological data. Each dose level would enroll an additional 6–9 patients.

### Clinical assessments

The severity of AEs was assessed per NCI-CTCAE version 5.0. AEs were recorded from the first dose of the study drug until 90 days after the last dose of the study drug or initiation of new antitumor therapy. Laboratory examinations included hematology tests, blood chemistry tests, cardiac function tests, urinalysis, coagulation tests, and thyroid function tests. Efficacy assessments according to RECIST version 1.1 were performed at baseline, every 6 weeks within 36 weeks after initial administration of the study drugs, then every 9 weeks until disease progression or initiation of subsequent antitumor therapy. Magnetic resonance imaging or 18 F-fluorodeoxyglucose positron emission tomography could be used when enhanced contrast computed tomography (CT) examination was not feasible.

### PK and immunogenicity analysis in human

To evaluate PK parameters, serum concentrations of JS007 were measured using a validated ELISA method. Samples were collected on Cycle 1 and Cycle 4 before infusion, then at 1 h (± 5 min), 6 h (± 15 min), 24 h (± 1 h), 48 h (± 2 h), 168 h (± 8 h), 336 h (± 12 h), and 504 h (± 12 h) after treatment. PK parameter values of an individual patient were derived by non-compartmental methods using a validated PK analysis program (Phoenix WinNonlin version 8.4, Certara, Princeton, NJ, USA). Anti-JS007 antibodies in human serum were detected utilizing a rigorously validated Bridging Electrochemiluminescence Immunoassay methodology. Samples were obtained within an hour before the administration of Cycle 1 through Cycle 4, 6, 8, 12, and 16, and then before the administration of every eight subsequent cycles. Post-treatment sample collection was planned for the end of the treatment course, with optional collections at 30 (± 7) days and 90 (± 7) days after the final treatment.

### Statistical analysis

The conventional 3 + 3 design was employed to determine the number of patients enrolled at 5 dose levels in the dose escalation phase, and 6–9 patients were enrolled at each dose level in the dose expansion phase. The full analysis set comprised all patients who received at least one dose of JS007 and had follow-up data after administration. The response evaluable analysis set comprised all patients who received at least one dose of JS007 with an adequate baseline tumor assessment and had undergone at least 1 appropriate tumor response assessment. The safety analysis set comprised all patients who received at least one dose of JS007. DLT analysis set comprised all patients who received at least 80% of the planned dose of JS007 and completed DLT observation, or patients who experienced DLT in the DLT observation window after administration. PK analysis set comprised all patients who received JS007 and had at least one PK evaluable data after the administration of JS007. The ADA analysis set comprised all patients who received at least one dose of JS007 and had evaluable ADA data after the administration of JS007. A two-sided 95% confidence interval (CI) of ORR and DoR was computed using the Exact (Clopper-Pearson) method by dose group. PFS and OS were estimated by the Kaplan-Meier method with 95% CIs calculated based on log-log transformation.

## Results

### In vitro activities of JS007

**Fig. 1:**
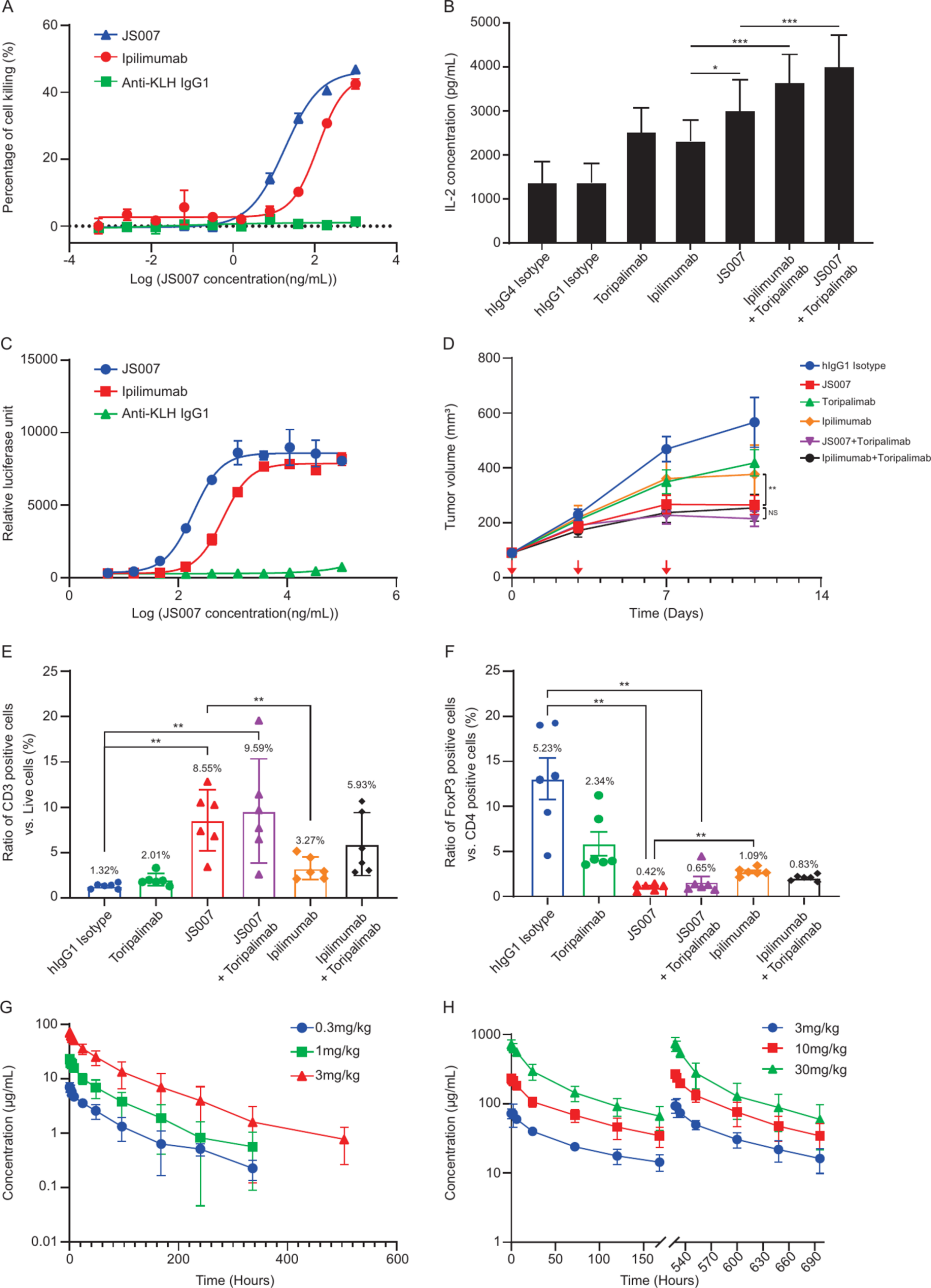
Preclinical characterization of JS007 in cell lines, mouse models and NHPs. **A**: Direct cytotoxicity test. **B**: IL-2 release levels of JS007 alone and in combination with toripalimab using the SEB model. (*P < 0.05; ***P < 0.001;). **C**: ADCC activation test. **D**: Anti-tumor effects of JS007 in B-hPD-1/hCTLA-4 mice MC38 xenograft model (**P < 0.01,* NS: no significance*). **E**: Infiltration of CD3^+^ cell in tumor microenvironment by TIL analysis (**P < 0.01). **F**: Infiltration of Treg cell in tumor microenvironment by TIL analysis (**P < 0.01). **G**: Serum concentration-time curve following a single intravenous infusion of 0.3, 1, and 3 mg/kg JS007 into Rhesus monkeys. The last time point of PK blood sampling is 1008 h, yet only samples of 3 mg/kg group can be detected with JS007 concentrations on 504 h, and all samples are below limit quantitative (BLQ) at 672, 840, and 1008 h. H: Serum concentration-time curve following repeated intravenous infusion of 3, 10 and 30 mg/kg JS007 after Day1 and Day22 administration

The binding affinity of JS007 was ~ 80 times higher than that of ipilimumab and the affinity of JS007 with various Fc receptors was comparable to ipilimumab (Table 1). In the direct cytotoxicity test, both JS007 and ipilimumab induced PBMC-mediated killing of the target cells in a concentration-dependent manner (Fig. 1A). The EC_50_ value was 18.37 ng/mL for JS007, ~ 6.6 folds of ipilimumab (120.9 ng/mL). The release of IL-2 from PBMCs was significantly increased by both JS007 and ipilimumab when combined with toripalimab (Fig. 1B). JS007 showed more potency in T cell activation than ipilimumab either monotherapy (*P* < 0.05) or combined with toripalimab, although a significant *P* value was not reached in the combination group. ADCC activation test showed that both JS007 and ipilimumab triggered FcγRIIIa-mediated fluorescent signal in a concentration-dependent manner. The EC_50_ value was 187 ng/mL for JS007 which was about 3.5 folds of ipilimumab (658 ng/mL, Fig. 1C).

**Table 1:** JS007 binding affinity to CTLA-4 protein and Fc receptor by Biacore

Binding Protein on Biacore	Antibody	Ka (1/Ms)	Kd (1/s)	KD (M)	Fold of Ipilimumab/JS007
Recombinant human CTLA4 protein	Ipilimumab	8.68 × 10^4^	1.39 × 10^− 3^	1.61 × 10^− 8^	78.15
	JS007	8.18 × 10^5^	1.69 × 10^− 4^	2.06 × 10^− 10^	
FcγRIIIa (CD16a) V176	Ipilimumab	1.90 × 10^5^	1.57 × 10^− 2^	8.27 × 10^− 8^	1.06
	JS007	1.52 × 10^5^	1.19 × 10^− 2^	7.80 × 10^− 8^	
FcγRIIIa (CD16a) F176	Ipilimumab	8.22 × 10^5^	5.83 × 10^− 2^	7.09 × 10^− 7^	1.70
	JS007	1.35 × 10^5^	5.62 × 10^− 2^	4.17 × 10^− 7^	
FcγRIIa (CD32a) R167	Ipilimumab	N/A	N/A	3.77 × 10^− 6^	0.85
	JS007	N/A	N/A	4.45 × 10^− 6^	
FcγRI (CD64)	Ipilimumab	6.70 × 10^5^	1.30 × 10^− 3^	1.94 × 10^− 9^	0.98
	JS007	6.48 × 10^5^	1.28 × 10^− 3^	1.97 × 10^− 9^	
FcRn	Ipilimumab	1.17 × 10^6^	8.21 × 10^− 2^	7.05 × 10^− 8^	1.24
	JS007	1.58 × 10^6^	8.99 × 10^− 2^	5.70 × 10^− 8^	

FcγRIIIa: two subtypes of Fc receptor type IIIa, V176, and F176, termed as cluster of differentiation 16a (CD16a), were surface markers of NK cells. FcγRIIa: Fc receptor type IIa, termed as cluster of differentiation 32a (CD32a), was a surface marker of monocytes or macrophages. FcγRI: Fc receptor type I, termed as cluster of differentiation 64 (CD64), mainly expressed on the basophils or mast cells. FcRn: neonatal Fc receptor, regulates the recycling of proteins

*Ka* association rate constant, *Kd* dissociation rate constant; Equilibrium constant KD = Kd/Ka

### In vivo antitumor efficacy of JS007

In the humanized mice MC38 xenograft model, the TGI ratio in the JS007 group was significantly higher than those in the ipilimumab group (63.3% vs. 40.0%, *P* < 0.01, Fig. 1D). Combined with toripalimab increased the TGI ratio of both JS007 and ipilimumab, and JS007 plus toripalimab group showed a numerically higher TGI ratio than ipilimumab plus toripalimab group (73.8% vs. 65.5%, *P *> 0.05). The TIL analysis demonstrated that JS007 induced a higher infiltration of CD3^+^ T cells compared with ipilimumab, evidenced by an increased percentage of CD3^+^ T cells within the live cell population (8.55% vs. 3.27%, *P* < 0.01, Fig. 1E). Additionally, a decrease in the percentage of Foxp3^+^ Treg cells was detected (0.42% vs. 1.09%, *P* < 0.01, Fig. 1F). T cell infiltration and Treg cell depletion mediated by JS007 and ipilimumab were both increased when combined with toripalimab.

### PK profiles in NHP models

In the single-dose experiment, the exposure of JS007 increased proportionally in a dose-dependent pattern (Fig. 1G, Table S1). The clearance rate was 0.78–0.96 mL/h/kg and the T_1/2_ was 60.7–70.8 h. No gender difference in the main PK parameters at each dose level was observed. In the repeat-dose experiment, a dose-proportional increase of JS007 exposure (C_max_ and AUC_0 − 168 h_) was also observed at both the first and the fourth dosing (Fig. 1H, Table S2). No blood accumulation was detected after four repeated doses compared to the initial dose. For the toxicological and pathological examination, JS007 of 30 mg/kg caused an animal death after multiple doses because of systemic inflammation and gastrointestinal toxicity, the expected systemic inflammations in 3 and 10 mg/kg groups were mild and recoverable after 4 weeks of recovery. Thus, the 10 mg/kg was determined as the HNSTD.

### Clinical characteristics of patients

From November 17, 2021, to October 9, 2023, a total of 28 patients were enrolled with 15 patients in the dose escalation phase and 13 patients in the dose expansion phase (Fig. S1). Demographic and baseline disease characteristics of patients are summarized in Table 2. A majority of the patients were male (71.4%) and had an ECOG PS of 0 (64.3%). The median age of patients was 57.8 (range 44.6–72.8) years. Twelve (42.8%) patients experienced ≥ 3 lines of prior treatment. The tumor types of patients included gastric cancer (*n* = 6), lung cancer (*n* = 4), esophageal cancer (*n* = 3), colon cancer, endometrial cancer, hepatic cancer, and pancreatic cancer (*n* = 2 each).

**Table 2:** Demographic and baseline disease characteristics (full analysis set)

	0.03 mg/kg (*N* = 1) *n*%	0.3 mg/kg (*N* = 3) *n*%	1 mg/kg (*N* = 5) *n*%	3 mg/kg (*N* = 9) *n*%	10 mg/kg (*N* = 10) *n*%	Total (*N* = 28) *n*%
Age, years						
Median (range)	57.00 (57.0–57.0)	52.60 (49.2–58.0)	59.30 (44.6–63.0)	60.90 (46.6–72.8)	57.40 (44.6–69.1)	57.80 (44.6–72.8)
Gender						
Male	0	2 (66.7)	5 (100)	6 (66.7)	7 (70.0)	20 (71.4)
Female	1 (100)	1 (33.3)	0	3 (33.3)	3 (30.0)	8 (28.6)
ECOG PS						
0	1 (100)	3 (100)	3 (60.0)	5 (55.6)	6 (60.0)	18 (64.3)
1	0	0	2 (40.0)	4 (44.4)	4 (40.0)	10 (35.7)
Prior lines of therapy						
1	0	0	3 (60.0)	1 (11.1)	3 (30.0)	7 (25.0)
2	1 (100)	2 (66.7)	1 (20.0)	3 (33.3)	2 (20.0)	9 (32.1)
3	0	0	0	5 (55.6)	4 (40.0)	9 (32.1)
≥ 4	0	1 (33.3)	1 (20.0)	0	1 (10.0)	3 (10.7)
Initial diagnosis						
Gastric cancer	0	1 (33.3)	0	3 (33.3)	2 (20.0)	6 (21.4)
Lung cancer	0	0	2 (40.0)	1 (11.1)	1 (10.0)	4 (14.3)
Oesophageal carcinoma	0	0	1 (20.0)	0	2 (20.0)	3 (10.7)
Colon cancer	0	0	0	1 (11.1)	1 (10.0)	2 (7.1)
Endometrial cancer	0	0	0	0	2 (20.0)	2 (7.1)
Hepatic cancer	0	1 (33.3)	1 (20.0)	0	0	2 (7.1)
Pancreatic carcinoma	1 (100.0)	0	0	0	1 (10.0)	2 (7.1)
Bile duct cancer	0	0	1 (20.0)	0	0	1 (3.6)
Bladder cancer	0	0	0	1 (11.1)	0	1 (3.6)
Breast cancer	0	0	0	1 (11.1)	0	1 (3.6)
Cervix carcinoma	0	0	0	0	1 (10.0)	1 (3.6)
Malignant neoplasm of ampulla of Vater	0	0	0	1 (11.1)	0	1 (3.6)
Nasopharyngeal cancer	0	1 (33.3)	0	0	0	1 (3.6)
Renal cancer	0	0	0	1 (11.1)	0	1 (3.6)

*ECOG PS* Eastern Cooperative Oncology Group performance status

### Safety

All 28 enrolled patients were included in safety analysis. The median treatment duration was 6.6 (range 3.0-66.9) weeks, and the median number of treatment cycles was 2 (range 1–9). No DLT was observed across all 5 dose levels, and the MTD did not reach. Dose levels of 3 mg/kg and 10 mg/kg were selected for the dose expansion phase. The treatment-emergent adverse event (TEAE) rate and treatment-related adverse event (TRAE) rate were 92.9% and 82.1%, respectively (Table 3). Grade ≥ 3 TEAEs occurred in 10 patients (35.7%, Table S3). Grade ≥ 3 TRAEs rate was 28.6% (8/28) and alanine aminotransferase increase (*n* = 2, 7.1%) was the most common one (Table S4). SAEs were reported in 6 (21.4%) patients, and 4 (14.3%) patients experienced treatment-related SAEs including ascites, diarrhea, asthenia, herpes zoster, and pulmonary embolism (*n* = 1, 3.6% each). Treatment delays due to TEAEs occurred in 10 (35.7%) patients, and 4 (14.3%) of them were related to study treatment. Permanent discontinuation of JS007 due to treatment-related lymph nodes pain was reported by 1 patient (3.6%) in 3 mg/kg group. Two (7.1%) patients reported Grade 1 or 2 irAEs including hyperthyroidism, hypothyroidism, amylase increase, lipase increase, interstitial lung disease, and pruritus. No AE-related death was reported.

**Table 3:** Overview of treatment-emergent adverse events (safety analysis set)

	0.03 mg/kg (*N* = 1) *n* (%)	0.3 mg/kg (*N* = 3) *n* (%)	1 mg/kg (*N* = 5) *n* (%)	3 mg/kg (*N* = 9) *n* (%)	10 mg/kg (*N* = 10) *n* (%)	Total (*N* = 28) *n* (%)
TEAEs	1 (100.0)	2 (66.7)	5 (100.0)	9 (100.0)	9 (90.0)	26 (92.9)
Treatment-related	1 (100.0)	2 (66.7)	3 (60.0)	8 (88.9)	9 (90.0)	23 (82.1)
Grade ≥ 3 TEAE	1 (100.0)	2 (66.7)	1 (20.0)	4 (44.4)	2 (20.0)	10 (35.7)
Treatment-related	1 (100.0)	1 (33.3)	1 (20.0)	4 (44.4)	1 (10.0)	8 (28.6)
Any TEAEs leading to drug interrupted	0	0	0	0	1 (10.0)	1 (3.6)
Treatment-related	0	0	0	0	1 (10.0)	1 (3.6)
Any TEAEs leading to drug delayed	1 (100.0)	1 (33.3)	2 (40.0)	5 (55.6)	1 (10.0)	10 (35.7)
Treatment-related	0	0	1 (20.0)	2 (22.2)	1 (10.0)	4 (14.3)
Any TEAEs leading to drug permanently discontinued	0	0	0	1 (11.1)	0	1 (3.6)
Treatment-related	0	0	0	1 (11.1)	0	1 (3.6)
Infusion reactions	0	0	0	0	1 (10.0)	1 (3.6)
irAEs	0	1 (33.3)	0	0	1 (10.0)	2 (7.1)
AESI	0	1 (33.3)	0	0	0	1 (3.6)
Treatment-related	0	1 (33.3)	0	0	0	1 (3.6)
SAE	0	1 (33.3)	1 (20.0)	3 (33.3)	1 (10.0)	6 (21.4)
Treatment-related	0	0	1 (20.0)	3 (33.3)	0	4 (14.3)
Any TEAEs leading to death	0	0	0	0	0	0
Treatment-related	0	0	0	0	0	0

*AESI* adverse event of special interest, *irAE* immune-related TEAE, *SAE* serious adverse event, *TEAE* treatment-emergent adverse event

### PK and immunogenicity in human

**Fig. 2:**
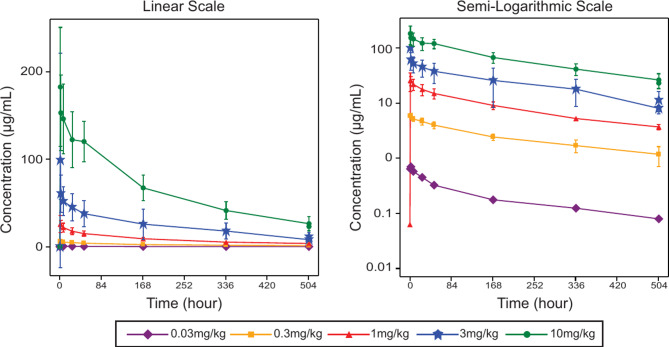
Mean concentration-time profiles of JS007 for 0.03 ~ 10 mg/kg dose cohorts

In the dose range from 0.03 to 10 mg/kg, patients administered with single dose of JS007 reached peak serum levels within 1.55 ~ 2.05 h of administration. Serum exposure to JS007 demonstrated a dose-dependent escalation (Fig. 2). Following a single administration, the mean values for C_max_ of JS007 were 74.5 µg/mL at 3 mg/kg dose and 180 µg/mL at 10 mg/kg dose with corresponding AUC_0 − tau_ of 11,300 h·µg/mL and 31,200 h·µg/mL, respectively. And the T_1/2_ of 0.03 ~ 10 mg/kg spanned 9.44 to 12.2 days (Table 4). The limited collection of pharmacokinetic samples after multiple administration affected the calculation of pharmacokinetic parameters (Data not shown). Steady-state concentrations appeared to have been reached by Cycle 4. Among the 26 patients assessed, 2 (7.69%) patients receiving the 3 mg/kg dose displayed treatment-induced ADA. Of these two patients, the median time to the earliest detection of ADA positivity was 51.43 days after the initial dose. No treatment-boosted ADA positivity was observed in this study (Table 5).

**Table 4:** Summary statistics for single-dose pharmacokinetic parameters of JS007 in the clinical trial

Parameters^*^(Unit)	0.03 mg/kg	0.3 mg/kg	1 mg/kg	3 mg/kg	10 mg/kg
	(*N* = 1)	(*N* = 3)	(*N* = 5 or 2)	(*N* = 9 or 7 or 6)	(*N* = 10 or 9)
C_max_ (µg/mL)	0.7	5.99 (6.5%)	26.8 (30.7%)	74.5 (84.8%)	180 (32.4%)
T_max_ (h)	1.55 (1.55,1.55)	1.53 (1.00,2.18)	2.05 (1.07,2.12)	1.98 (0.93,2.27)	2.00 (1.03,7.50)
AUC_0 − tau_ (h*µg/mL)	92.9	1170 (16%)	4380 (14.2%)	11,300 (51.6%)	31,200 (17.6%)
AUC_0 − t_ (h*µg/mL)	92.9	1180 (16.2%)	4370 (14.5%)	9950 (40.9%)	31,300 (17.4%)
AUC_0−∞_ (h*µg/mL)	126	1600 (20%)	5870 (0.6%)	15,300 (54.9%)	39,900 (16.3%)
CL (L/day)	0.313	0.287 (31%)	0.306 (4.3%)	0.268 (46.3%)	0.365 (22.9%)
V_ss_ (L)	4.91	4.50 (32.8%)	4.48 (38%)	4.17 (34.9%)	4.84 (25.3%)
T_1/2_ (day)	12.2	10.7 (7.9%)	11.1 (31.6%)	11.0 (26%)	9.44 (25.6%)
MRT_0−∞_ (day)	15.7	15.7 (9%)	14.6 (33.2%)	15.6 (20.8%)	13.2 (25.2%)
%AUCex (%)	26.6	26.3 (12.8%)	24.1 (44.3%)	27.6 (28.6%)	19.8 (41.2%)

^*^ Arithmetic Geometric mean (Geometric CV%) is reported for all parameters except T_max_, which reports median (range)

*C*_*ma*x_ maximum observed concentration

*T*_*max*_ time of maximum observed concentration

*AUC*_*0 − tau*_ area under the curve from time 0 extrapolated to dose interval

*AUC*_*0 − t*_ area under the curve from time 0 to the last measurable concentration

*AUC*_*0−∞*_ area under the curve from time 0 extrapolated to infinity

*CL* clearance

*V*_*ss*_ volume of distribution at steady state after single intravenous administration

*T*_*1/2*_ apparent terminal phase half-life of elimination

*MRT*_*0−∞*_ mean residence time (MRT) extrapolated to infinity

*%AUCex* area under the curve from the time of the last measurable concentration to infinity, as a percentage of the curve extrapolated to infinity

The parameters cannot be calculated due to the absence of end elimination phase samples in some subjects ranging from 1 to 10 mg/kg

**Table 5:** Immunogenicity summary by JS007 dose cohorts

	0.03 mg/kg	0.3 mg/kg	1 mg/kg	3 mg/kg	10 mg/kg	Total
	(*N* = 1)	(*N* = 3)	(*N* = 5)	(*N* = 8)	(*N* = 9)	(*N* = 26)
	n (%)	n (%)	n (%)	n (%)	n (%)	n (%)
ADA-Negative ^a^	1 (100.00)	3 (100.00)	5 (100.00)	6 (75.00)	9 (100.00)	24 (92.31)
ADA-Positive ^b^	0	0	0	2 (25.00)	0	2 (7.69)
Treatment-induced ADA ^c^	0	0	0	2 (25.00)	0	2 (7.69)
Treatment-boosted ADA ^d^	0	0	0	0	0	0
Duration of First Positive (Days)						
n	NA	NA	NA	2	NA	NA
Median (Min, Max)	NA	NA	NA	51.43 (27.93,74.93)	NA	NA

^a^ ADA-Negative: Subjects without a treatment-induced or treatment-boosted ADA-positive sample during the treatment or follow-up observation period

^b^ ADA-Positive: Subjects with at least one treatment-induced or treatment-boosted ADA-positive sample at any time during the treatment or follow-up observation period

^c^ Treatment-induced ADA: ADA developed de novo (seroconversion) following biologic drug administration

^d^ Treatment-boosted ADA: Pre-existing ADA that was boosted to a higher level following biologic drug administration

### Efficacy

**Fig. 3:**
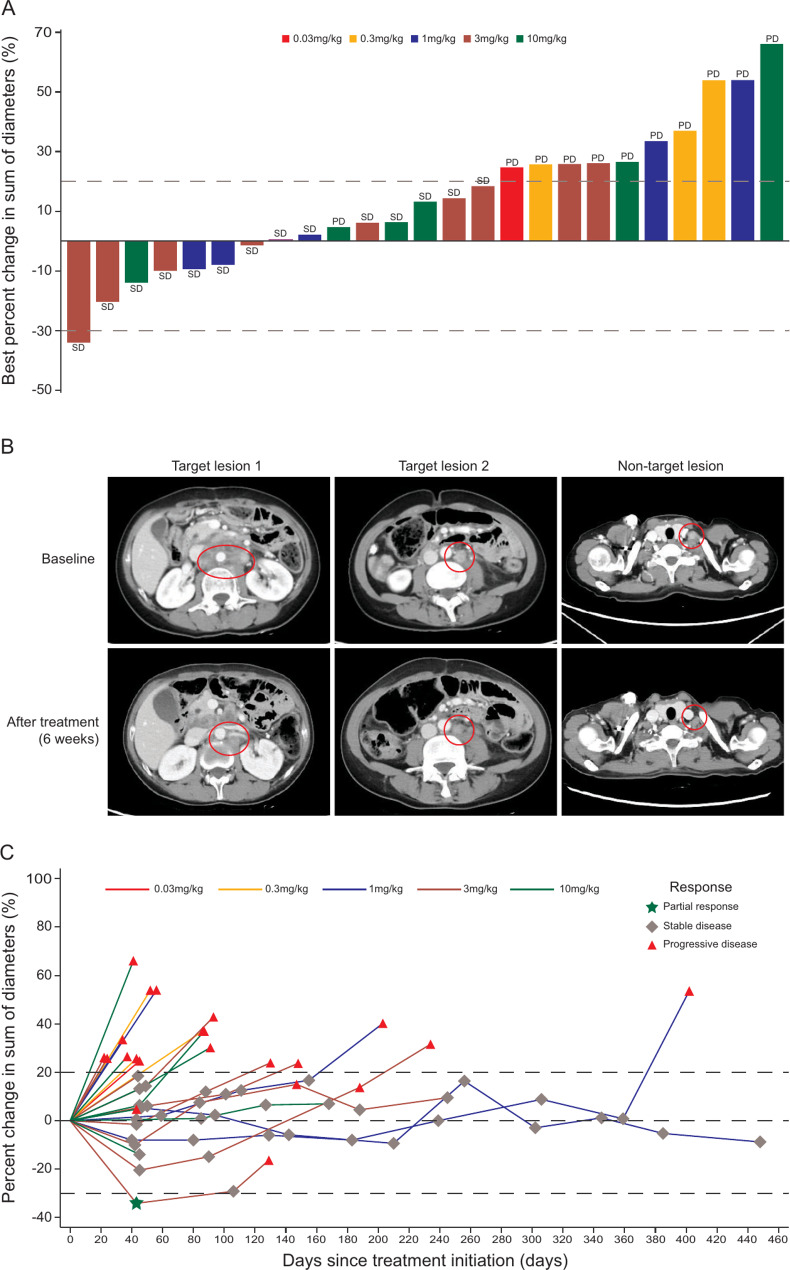
Treatment efficacy of JS007. **A**: Waterfall plot for best percent change in sum of diameters of target lesions from baseline (full analysis set). **B**: Radiological images of target lesions over time in a patient with gastric cancer. **C**: Spider plot for percent change in sum of diameters of target lesions from baseline (full analysis set)

Among all 28 patients, 25 had post-baseline tumor assessments. As of the data cutoff date, the median follow-up was 6.7 months, and 20 patients had disease progression; 10 patients died. Fourteen (50%) patients achieved confirmed SD, and no confirmed objective response was observed (Fig. 3A, Table S5). A 46-year-old female patient with gastric cancer in 3 mg/kg group progressed after 3 prior lines of therapy achieved an unconfirmed PR, with a 34.1% reduction of target lesion after 6 weeks of JS007 treatment (Fig. 3B). The confirmed DCR was 50.0% (95% CI 30.7–69.4). Median PFS was 2.8 (95% CI 1.4–4.3) months, and median OS was 14.7 (95% CI 8.4-not reached) months (Fig. 3C, Table S5). Eight (57.1%) patients with confirmed SD experienced a duration of non-progression disease over 4 months. One NSCLC patient with prior immunotherapy and one esophagus squamous cell carcinoma (ESCC) patient without prior immunotherapy achieved durable disease control of 14.7 and 13.2 months, respectively.

## Discussion

Here, we presented the preclinical data and results of the first-in-human clinical study of JS007 in patients with advanced solid tumors. The comprehensive preclinical results demonstrated excellent binding affinity, immune response activation, and antitumor activity of JS007. In the clinical study, the MTD was not reached even at the 10 mg/kg dose level. The rate of Grade ≥ 3 TRAEs was 28.6%, and no unexpected irAE was reported. Effective disease control and long-term survival in these heavily treated cancer patients indicated the encouraging antitumor efficacy of JS007.

JS007, a humanized IgG1 mAb, was generated based on hybridoma technology aimed to improve its affinity for CTLA-4. The potent binding affinity between JS007 and CTLA-4 as well as the blocking effect on CTLA-4/B7 interaction was attributed to its unique structure revealed in our previous study [22]. In this study, the superior binding potency of JS007 significantly improved the in vitro killing effect by PBMCs, as well as a superior in vivo antitumor efficacy compared with the reference mAb. In addition to the direct effects on effector T cells, JS007 demonstrated a more effective depletion of Treg cells in vivo. The infiltration of Treg cells in tumor microenvironment (TME) is a critical factor affecting the efficacy of ICI treatment. CTLA-4 plays a vital role in maintaining Treg cells stability. Du, et al. demonstrated that the therapeutic effect of anti-CTLA4 mAb required antibody-mediated depletion of Tregs cells in the TME [24]. Moreover, eliminating Treg cells in the TME is now a key objective in developing second-generation anti-CTLA-4 antibody. Therefore, the in vivo results of our study demonstrated a promising antitumor effect of JS007.

Based on these preclinical data, the phase 1a, first-in-human clinical trial was launched. The safety profile of JS007 monotherapy in this trial was excellent and comparable to that of CTLA-4 antibodies previously reported, regardless of the enrollment of patients with heterogeneous tumor types and multiple prior lines of therapy. No unexpected AEs were observed in this study. The most common Grade ≥ 3 TRAEs was increased alanine aminotransferase which occurred in 2 patients. Ipilimumab is the most widely used CTLA-4 antibody currently, and the rate of Grade ≥ 3 TRAEs of its monotherapy was about 30%. Patients treated with 10 mg/kg of ipilimumab showed a higher incidence of Grade ≥ 3 TRAEs compared with 3 mg/kg [6, 25]. Tremelimumab is the other approved CTLA-4 antibody, and its Grade ≥ 3 TEAE rate reported in the phase 3 trial with advanced melanoma was 52% [17]. Diarrhea was the most common Grade ≥ 3 TRAE of both ipilimumab and tremelimumab which was reported by only 1 patient in the present study.

The antitumor activity of anti-CTLA-4 antibody monotherapy has been demonstrated in melanoma. Although the sample size was limited, a promising treatment efficacy of JS007 was observed, with the confirmed DCR being 50% in this study. In a phase 1 trial, a total of 25 Chinese patients with recurrent malignant melanoma or nasopharyngeal carcinoma received ipilimumab, only 3 patients (12%) achieved SD [26]. In another early-stage trial, 34 patients with advanced melanoma were treated with tremelimumab monotherapy. The ORR and DCR were 10.3% and 20.5%, respectively [27]. In this study, more than half of the enrolled patients had previously treated gastrointestinal adenocarcinoma, which was not as sensitive as melanoma to immunotherapy. Nevertheless, long-term survival was achieved in two patients, one with NSCLC and one with ESCC, who experienced durable disease control of 14.7 and 13.2 months, respectively.

Our results showed the preliminary antitumor effect of JS007 as monotherapy in patients with previously heavily treated advanced solid tumors and the MTD of its monotherapy was not defined. However, the combination of anti-CTLA-4 antibodies and PD-1/PD-L1 inhibitors, irrespective of a higher risk of irAEs, has been demonstrated as a more effective strategy than anti-CTLA-4 antibody monotherapy in solid tumors [4, 6–15, 28, 29]. In this study, a synergistic antitumor effect of JS007 combined with toripalimab, a PD-1 inhibitor, was observed in preclinical study, including effector T cell activation and Treg cell depletion. A similar PK profile of JS007 in the NHP model and patients with ipilimumab at equivalent dosages suggested the feasibility of JS007 combined with PD-1 antibodies [6, 30]. In the future study, the efficacy and safety of JS007 combined with other ICI are worth assessing in selected indications.

There were potential limitations of our study. First, the sample size was limited. By adopting a modified 3 + 3 dose escalation design and following the dose expansion phase, the safety profiles of JS007 were fully investigated at different dose levels which met the primary endpoint of this study. In future study with a larger sample size, JS007 combined with toripalimab should be assessed. Second, only Chinese patients were enrolled. Although the PK profile of ipilimumab was reported as ethnically insensitive, the ethical difference of JS007 should be assessed in future investigations.

## Conclusions

In this study, JS007 was well tolerated and showed encouraging antitumor activities in heavily treated patients with advanced solid tumors. Pharmacokinetic profiles of JS007 were determined, which warrants future study of JS007 combination therapy.

## Electronic supplementary material

Below is the link to the electronic supplementary material.

Supplementary Material 1

Supplementary Material 2

## Data Availability

The datasets used and/or analyzed during the current study are available from the corresponding author upon reasonable request.

## Abbreviations

ADA: Antidrug antibody
ADCC: Antibody-dependent cellular cytotoxicity
AE: Adverse event
AUC: Area under the plasma drug concentration-time curve
CI: Confidence interval
CL: Clearance
C_max_: Maximum observed concentration
CT: Computed tomography
CTLA-4: Cytotoxic T lymphocyte-associated antigen-4
CR: Complete response
DCR: Disease control rate
DLT: Dose-limiting toxicity
DoR: Duration of response
ECD: Extracellular domain
ECOG PS: Eastern Cooperative Oncology Group performance status
ELISA: Enzyme-linked Immunosorbent Assay
FDA: Food and Drug Administration
HCC: Hepatocellular carcinoma
HNSTD: Highest non-severely toxic dose
IACUC: Institutional Animal Care and Use Committee
ICIs: Immune checkpoint inhibitors
irAE: Immune-related adverse event
LDH: Lactate dehydrogenase
mAb: Monoclonal antibody
MTD: Maximum tolerated dose
NCI-CTCAE: National Cancer Institute Common Terminology Criteria for Adverse Events
NHP: Non-human primate
NSCLC: Non-small cell lung cancer
ORR: Objective response rate
OS: Overall survival
PBMC: Peripheral blood mononuclear cell
PFS: Progression-free survival
PK: Pharmacokinetics
PD-1: Programmed cell death protein 1
PD-L1: Programmed cell death ligand 1
PR: Partial response
RECIST: Response Evaluation Criteria in Solid Tumors
SAE: Serious adverse event
SD: Stable disease
SEB: Staphylococcal enterotoxin B
TEAE: Treatment-emergent adverse event
TGI: Tumor growth inhibition
TIL: tumor-infiltrated cell
TME: Tumor microenvironment
TRAEs: Treatment-related adverse event
Treg: T regulatory
Tv: Tumor volume
T_1/2_: Elimination half-life
