# Supplementary figures and images for: Preclinical investigations and a first-in-human phase 1a trial of JS007, a novel anti-CTLA-4 antibody, in patients with advanced solid tumors
Source: Exp Hematol Oncol. 2024 Oct 1;13:98. doi: 10.1186/s40164-024-00567-7 (PMC11443874; doi:10.1186/s40164-024-00567-7)

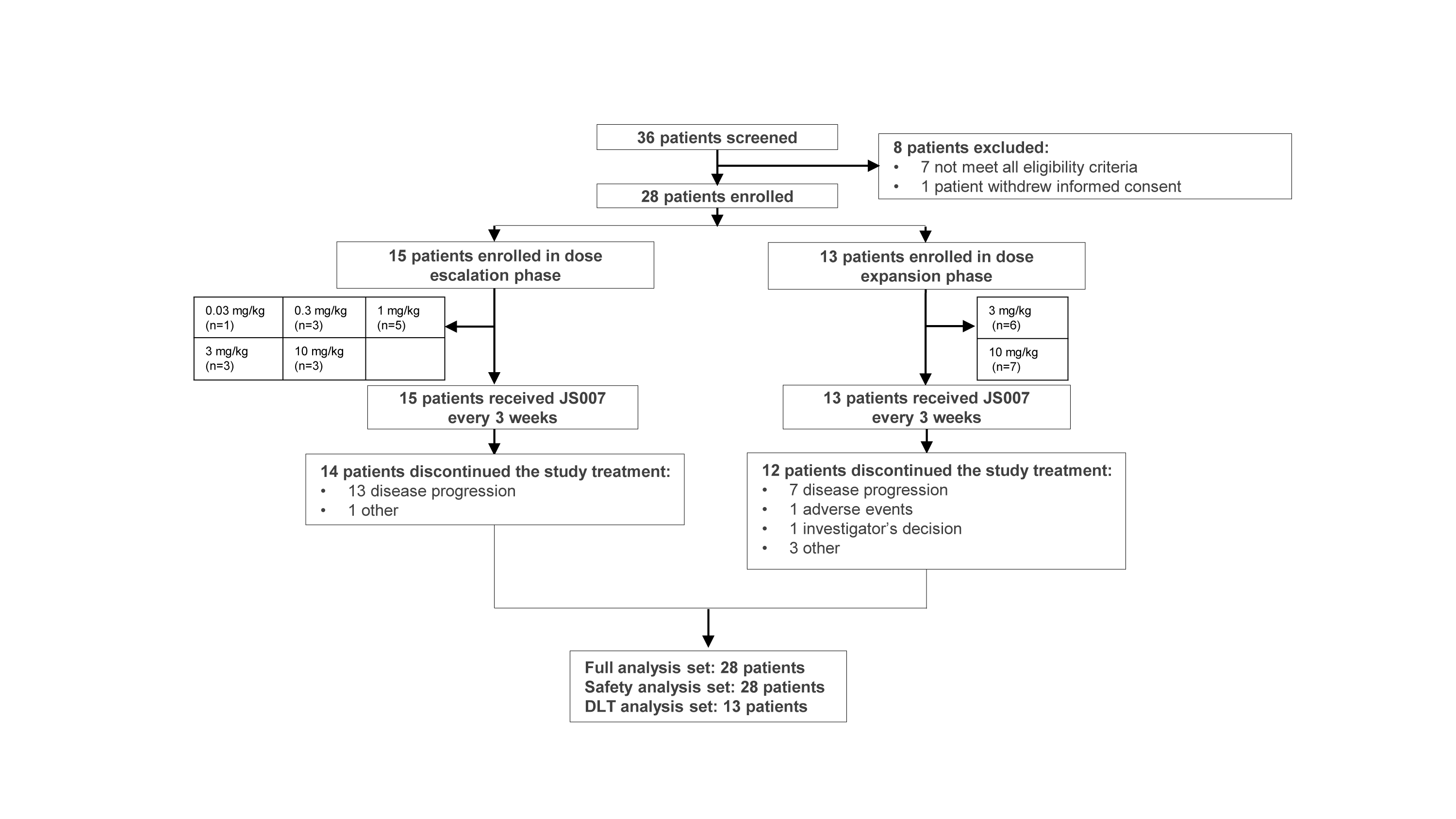

Supplement: Supplementary file 1 — Supplementary Material 1 [file 40164_2024_567_MOESM1_ESM.tif]
