# Supplementary material for: Preclinical investigations and a first-in-human phase 1a trial of JS007, a novel anti-CTLA-4 antibody, in patients with advanced solid tumors
Source: Exp Hematol Oncol. 2024 Oct 1;13:98. doi: 10.1186/s40164-024-00567-7 (PMC11443874; doi:10.1186/s40164-024-00567-7)
Supplement: Supplementary file 2 — Supplementary Material 2 [file 40164_2024_567_MOESM2_ESM.docx]

**Supplementary Table S1. Single-dose pharmacokinetic data of monkeys after intravenous injection of JS007**

| Parameters | Units | **0.3 mg/kg (n=6)** | | | **1 mg/kg (n=6)** | | | **3 mg/kg group (n=6 or n=5)** | | |
| --- | --- | --- | --- | --- | --- | --- | --- | --- | --- | --- |
|  |  | **Mean** | **SD** | **CV%** | **Mean** | **SD** | **CV%** | **Mean** | **SD** | **CV%** |
| AUC__%Extrap_obs_ | % | 4.03 | 1.00 | 24.9 | 1.52 | 0.570 | 37.5 | 0.649 | 0.186 | 28.6 |
| AUC_INF_obs_ | h*μg/mL | 416 | 153 | 36.7 | 1220 | 503 | 41.2 | 4560 | 1730 | 37.9 |
| AUC_last_ | h*μg/mL | 399 | 147 | 36.9 | 1200 | 496 | 41.3 | 4150 | 1790 | 43.1 |
| C_0_ | μg/mL | 7.09 | 1.19 | 16.7 | 24.2 | 2.94 | 12.1 | 70.9 | 6.06 | 8.56 |
| Cl__obs_ | mL/h/kg | 0.800 | 0.264 | 33.0 | 0.957 | 0.428 | 44.7 | 0.780 | 0.426 | 54.6 |
| MRT_INF_obs_ | h | 80.0 | 35.3 | 44.2 | 76.1 | 30.3 | 39.8 | 81.6 | 35.3 | 43.3 |
| MRT_last_ | h | 68.0 | 30.0 | 44.1 | 70.4 | 28.6 | 40.6 | 74.2 | 38.6 | 52.0 |
| T_1/2_z_ | h | 62.8 | 33.4 | 53.2 | 60.7 | 20.0 | 32.9 | 70.8 | 22.0 | 31.1 |
| T_max_ | h | 0.375 | 0.250 | 0.500 | 0.250 | 0.250 | 0.500 | 0.500 | 0.250 | 0.500 |
| V_ss_obs_ | mL/kg | 57.0 | 12.4 | 21.8 | 63.5 | 7.66 | 12.1 | 56.9 | 6.73 | 11.8 |

*AUC__%Extrap_obs_* area under the curve from the time of the last measurable concentration to infinity, as a percentage of the curve extrapolated to infinity.

*AUC_INF_obs_* area under the curve from time 0 extrapolated to infinity.

*AUC_last_* area under the curve from time 0 to the last measurable (positive) concentration.

*C_0_* concentration at time 0.

*Cl__obs_* total body clearance observed.

*MRT_INF_obs_* mean residence time (MRT) extrapolated to Infinity.

*MRT_last_* mean residence time from time 0 to the time of the last measurable concentration.

*T_1/2_z_* apparent terminal phase half-life of elimination.

*T_max_* time of maximum observed concentration.

*V_ss_obs_* volume of distribution at steady state after single intravenous administration.

*SD* standard deviation.

*CV%* coefficient of variation.

N=5 for AUC_% Extrap, AUC_inf_, and CL in 3 mg/kg due to the value meeting the excluding criteria that estimated AUC _Extrap_obs_ 20% higher than the AUC_inf_obs_.

**Supplementary Table S2. Pharmacokinetic data of monkeys after 4-week repeated dosing of JS007 via intravenous injection**

| Species/Strain: Rhesus monkey | **Dosing Duration:**  **4 weeks** | | **Recovery period: 4 weeks** | | **Administration Route: Intravenous infusion** | | **Age: (Male) 3.1-4.4 years;**  **(Female) 3.0 to 3.5 years** | |
| --- | --- | --- | --- | --- | --- | --- | --- | --- |
| Dose (mg/kg) | **0** | | **3** | | **10** | | **30** | |
| Number of animals | 5 Males | 5 Females | 5 Males | 5 Females | 5 Males | 5 Females | 5 Males | 5 Females |
| Day1 (First Dosing) | | | | | | | | |
| C_max_ (µg/mL) | BQL | BQL | 76.582±14 | 84.312±31 | 216.069±29 | 249.264±48 | 681.429±51 | 759.454±181 |
| AUC_0-168h_ (µg*hr/mL) | BQL | BQL | 4735.0±587 | 4456.0±487 | 11812.0±2834 | 13655.6±1846 | 32417.5±4537 | 30376.7±8825 |
| Day22 (Fourth Dosing) | | | | | | | | |
| C_max_ (µg/mL) | BQL | BQL | 101.242±35 | 99.371±12 | 246.204±27 | 293.291±29 | 670.659±94 | 839.844±240 |
| AUC_0-168h_ (µg*hr/mL) | BQL | BQL | 6051.6±1493 | 5374.9±978 | 13448.7±2080 | 14954±4014 | 25297.2±11579 | 31934.8±8620 |

*AUC_0-168h_* area under curve from 0 hour to 168 hours

*BQL* below quantitation limit

C*max* maximal concentration

**Supplementary Table S3. Treatment-emergent adverse events occurring in ≥ 10% of patients of any grade or 1% of Grade ≥3 at different dose levels (safety analysis set)**

|  | **0.03 mg/kg (N=1)** | | **0.3 mg/kg (N=3)** | | **1 mg/kg (N=5)** | | | **3 mg/kg (N=9)** | | **10 mg/kg (N=10)** | | **Total (N=28)** | |
| --- | --- | --- | --- | --- | --- | --- | --- | --- | --- | --- | --- | --- | --- |
|  | Any grade | Grade ≥3 | Any grade | Grade ≥3 | | Any grade | Grade ≥3 | Any grade | Grade ≥3 | Any grade | Grade ≥3 | Any grade | Grade ≥3 |
| Number of patients with TEAEs or Grade ≥3 TEAEs | 1 (100) | 1 (100.0) | 2 (66.7) | 2 (66.7) | | 5 (100.0) | 1 (20.0) | 9 (100.0) | 4 (44.4) | 9 (90.0) | 2 (20.0) | 26 (92.9) | 10 (35.7) |
| Clinically reported sign and symptoms |  |  |  |  | |  |  |  |  |  |  |  |  |
| COVID-19 | 0 | 0 | 0 | 0 | | 2 (40.0) | 0 | 6 (66.7) | 1 (11.1) | 2 (20.0) | 0 | 10 (35.7) | 1 (3.6) |
| Pruritus | 0 | 0 | 0 | 0 | | 1 (20.0) | 0 | 3 (33.3) | 1 (11.1) | 4 (40.0) | 0 | 8 (28.6) | 1 (3.6) |
| Anaemia | 0 | 0 | 0 | 0 | | 0 | 0 | 3 (33.3) | 1 (11.1) | 2 (20.0) | 0 | 5 (17.9) | 1 (3.6) |
| Rash | 0 | 0 | 0 | 0 | | 1 (20.0) | 0 | 4 (44.4) | 0 | 0 | 0 | 5 (17.9) | 0 |
| Albuminuria | 0 | 0 | 0 | 0 | | 0 | 0 | 3 (33.3) | 0 | 2 (20.0) | 0 | 5 (17.9) | 0 |
| Nausea | 1 (100.0) | 0 | 0 | 0 | | 1 (20.0) | 0 | 2 (22.2) | 0 | 0 | 0 | 4 (14.3) | 0 |
| Hypoalbuminaemia | 0 | 0 | 0 | 0 | | 0 | 0 | 2 (22.2) | 0 | 2 (20.0) | 0 | 4 (14.3) | 0 |
| Vomiting | 1 (100.0) | 0 | 0 | 0 | | 0 | 0 | 2 (22.2) | 0 | 0 | 0 | 3 (10.7) | 0 |
| Nausea | 1 (100.0) | 0 | 0 | 0 | | 0 | 0 | 2 (22.2) | 0 | 0 | 0 | 3 (10.7) | 0 |
| Hypokalaemia | 0 | 0 | 1 (33.3) | 0 | | 1 (20.0) | 0 | 1 (11.1) | 1 (11.1) | 0 | 0 | 3 (10.7) | 1 (3.6) |
| Diarrhoea | 0 | 0 | 0 | 0 | | 0 | 0 | 1 (11.1) | 1 (11.1) | 1 (10.0) | 0 | 2 (7.1) | 1 (3.6) |
| Ascites | 0 | 0 | 0 | 0 | | 0 | 0 | 1 (11.1) | 1 (11.1) | 0 | 0 | 1 (3.6) | 1 (3.6) |
| Duodenal obstruction | 0 | 0 | 0 | 0 | | 0 | 0 | 0 | 0 | 1 (10.0) | 1 (10.0) | 1 (3.6) | 1 (3.6) |
| Gamma-glutamyltransferase increased | 0 | 0 | 1 (33.3) | 1 (33.3) | | 0 | 0 | 0 | 0 | 0 | 0 | 1 (3.6) | 1 (3.6) |
| Device related infection | 0 | 0 | 1 (33.3) | 1 (33.3) | | 0 | 0 | 0 | 0 | 0 | 0 | 1 (3.6) | 1 (3.6) |
| Hyponatraemia | 0 | 0 | 0 | 0 | | 0 | 0 | 1 (11.1) | 1 (11.1) | 0 | 0 | 1 (3.6) | 1 (3.6) |
| Bilirubin conjugated increased | 1 (100.0) | 1 (100.0) | 0 | 0 | | 0 | 0 | 0 | 0 | 0 | 0 | 1 (3.6) | 1 (3.6) |
| Asthenia | 0 | 0 | 0 | 0 | | 0 | 0 | 1 (11.1) | 1 (11.1) | 0 | 0 | 1 (3.6) | 1 (3.6) |
| Pulmonary embolism | 0 | 0 | 0 | 0 | | 1 (20.0) | 1 (20.0) | 0 | 0 | 0 | 0 | 1 (3.6) | 1 (3.6) |
| Laboratory Abnormalities |  |  |  |  | |  |  |  |  |  |  |  |  |
| Aspartate aminotransferase increased | 1 (100.0) | 1 (100.0) | 0 | 0 | | 1 (20.0) | 0 | 2 (22.2) | 0 | 4 (40.0) | 0 | 8 (28.6) | 1 (3.6) |
| Weight decreased | 0 | 0 | 0 | 0 | | 0 | 0 | 3 (33.3) | 0 | 5 (50.0) | 0 | 8 (28.6) | 0 |
| Alanine aminotransferase increased | 1 (100.0) | 0 | 1 (33.3) | 0 | | 1 (20.0) | 1 (20.0) | 1 (11.1) | 0 | 3 (30.0) | 1 (10.0) | 7 (25.0) | 2 (7.1) |
| Blood alkaline phosphatase increased | 1 (100.0) | 0 | 1 (33.3) | 0 | | 0 | 0 | 1 (11.1) | 0 | 1 (10.0) | 0 | 4 (14.3) | 0 |
| Weight increased | 0 | 0 | 0 | 0 | | 2 (40.0) | 0 | 1 (11.1) | 0 | 1 (10.0) | 0 | 4 (14.3) | 0 |
| Haemoglobin decreased | 1 (100.0) | 0 | 1 (33.3) | 0 | | 0 | 0 | 0 | 0 | 1 (10.0) | 0 | 3 (10.7) | 0 |
| Amylase increased | 0 | 0 | 0 | 0 | | 0 | 0 | 1 (11.1) | 1 (11.1) | 2 (20.0) | 0 | 3 (10.7) | 1 (3.6) |
| Blood bilirubin increased | 1 (100.0) | 1 (100.0) | 0 | 0 | | 0 | 0 | 1 (11.1) | 0 | 1 (10.0) | 0 | 3 (10.7) | 1 (3.6) |
| Lipase increased | 0 | 0 | 0 | 0 | | 1 (20.0) | 0 | 1 (11.1) | 1 (11.1) | 1 (10.0) | 0 | 3 (10.7) | 1 (3.6) |

*TEAE* treatment-emergent adverse event

**Supplementary Table S4. TRAEs occurring in ≥ 5% of patients of any grade or 1% of Grade ≥3 at different dose levels (safety analysis set)**

|  | **0.03 mg/kg (N=1)** | | **0.3 mg/kg (N=3)** | | **1 mg/kg (N=5)** | | **3 mg/kg (N=9)** | | **10 mg/kg (N=10)** | | **Total (N=28)** | |
| --- | --- | --- | --- | --- | --- | --- | --- | --- | --- | --- | --- | --- |
|  | Any grade | Grade ≥3 | Any grade | Grade ≥3 | Any grade | Grade ≥3 | Any grade | Grade ≥3 | Any grade | Grade ≥3 | Any grade | Grade ≥3 |
| Number of patients with TRAEs or Grade ≥3 TRAEs | 1 (100) | 1 (100.0) | 2 (66.7) | 1 (33.3) | 3 (60.0) | 1 (20.0) | 8 (88.9) | 4 (44.4) | 9 (90.0) | 1 (10.0) | 23 (82.1) | 8 (28.6) |

| Clinically reported sign and symptoms |  |  |  |  |  |  |  |  |  |  |  |  |
| --- | --- | --- | --- | --- | --- | --- | --- | --- | --- | --- | --- | --- |
| Pruritus | 0 | 0 | 0 | 0 | 1 (20.0) | 0 | 3 (33.3) | 1 (11.1) | 4 (40.0) | 0 | 8 (28.6) | 1 (3.6) |
| Rash | 0 | 0 | 0 | 0 | 1 (20.0) | 0 | 4 (44.4) | 0 | 0 | 0 | 5 (17.9) | 0 |
| Anaemia | 0 | 0 | 0 | 0 | 0 | 0 | 3 (33.3) | 1 (11.1) | 2 (20.0) | 0 | 5 (17.9) | 1 (3.6) |
| Albuminuria | 0 | 0 | 0 | 0 | 0 | 0 | 3 (33.3) | 0 | 2 (20.0) | 0 | 5 (17.9) | 0 |
| Nausea | 1 (100.0) | 0 | 0 | 0 | 1 (20.0) | 0 | 2 (22.2) | 0 | 0 | 0 | 4 (14.3) | 0 |
| Hypoalbuminaemia | 0 | 0 | 0 | 0 | 0 | 0 | 2 (22.2) | 0 | 1 (10.0) | 0 | 3 (10.7) | 0 |
| Hypokalaemia | 0 | 0 | 1 (33.3) | 0 | 1 (20.0) | 0 | 1 (11.1) | 1 (11.1) | 0 | 0 | 3 (10.7) | 1 (3.6) |
| Vomiting | 1 (100.0) | 0 | 0 | 0 | 0 | 0 | 2 (22.2) | 0 | 0 | 0 | 3 (10.7) | 0 |
| Diarrhoea | 0 | 0 | 0 | 0 | 0 | 0 | 1 (11.1) | 1 (11.1) | 1 (10.0) | 0 | 2 (7.1) | 1 (3.6) |
| Bilirubin conjugated increased | 1 (100.0) | 1 (100.0) | 0 | 0 | 0 | 0 | 0 | 0 | 0 | 0 | 1 (3.6) | 1 (3.6) |
| Gamma-glutamyltransferase increased | 0 | 0 | 1 (33.3) | 1 (33.3) | 0 | 0 | 0 | 0 | 0 | 0 | 1 (3.6) | 1 (3.6) |
| Ascites | 0 | 0 | 0 | 0 | 0 | 0 | 1 (11.1) | 1 (11.1) | 0 | 0 | 1 (3.6) | 1 (3.6) |
| Hyponatraemia | 0 | 0 | 0 | 0 | 0 | 0 | 1 (11.1) | 1 (11.1) | 0 | 0 | 1 (3.6) | 1 (3.6) |
| Asthenia | 0 | 0 | 0 | 0 | 0 | 0 | 1 (11.1) | 1 (11.1) | 0 | 0 | 1 (3.6) | 1 (3.6) |
| Pulmonary embolism | 0 | 0 | 0 | 0 | 1 (20.0) | 1 (20.0) | 0 | 0 | 0 | 0 | 1 (3.6) | 1 (3.6) |
| Laboratory Abnormalities |  |  |  |  |  |  |  |  |  |  |  |  |
| Aspartate aminotransferase increased | 0 | 0 | 0 | 0 | 1 (20.0) | 0 | 1 (11.1) | 0 | 4 (40.0) | 0 | 6 (21.4) | 0 |
| Alanine aminotransferase increased | 0 | 0 | 1 (33.3) | 0 | 1 (20.0) | 1 (20.0) | 0 | 0 | 3 (30.0) | 1 (10.0) | 5 (17.9) | 2 (7.1) |
| Weight decreased | 0 | 0 | 0 | 0 | 0 | 0 | 2 (22.2) | 0 | 3 (30.0) | 0 | 5 (17.9) | 0 |
| Amylase increased | 0 | 0 | 0 | 0 | 0 | 0 | 1 (11.1) | 1 (11.1) | 2 (20.0) | 0 | 3 (10.7) | 1 (3.6) |
| Blood bilirubin increased | 1 (100.0) | 1 (100.0) | 0 | 0 | 0 | 0 | 1 (11.1) | 0 | 1 (10.0) | 0 | 3 (10.7) | 1 (3.6) |
| Haemoglobin decreased | 1 (100.0) | 0 | 1 (33.3) | 0 | 0 | 0 | 0 | 0 | 1 (10.0) | 0 | 3 (10.7) | 0 |
| Lipase increased | 0 | 0 | 0 | 0 | 1 (20.0) | 0 | 1 (11.1) | 1 (11.1) | 1 (10.0) | 0 | 3 (10.7) | 1 (3.6) |
| Blood alkaline phosphatase increased | 0 | 0 | 1 (33.3) | 0 | 0 | 0 | 0 | 0 | 1 (10.0) | 0 | 2 (7.1) | 0 |
| Lymphocyte count decreased | 0 | 0 | 1 (33.3) | 0 | 0 | 0 | 0 | 0 | 1 (10.0) | 0 | 2 (7.1) | 0 |
| Weight increased | 0 | 0 | 0 | 0 | 0 | 0 | 1 (11.1) | 0 | 1 (10.0) | 0 | 2 (7.1) | 0 |

*TEAE* treatment-emergent adverse event, *TRAE* treatment-related TEAE

**Supplementary Table S5. Summary of tumor response**

|  | **0.03 mg/kg (N=1)** | **0.3 mg/kg (N=3)** | **1 mg/kg (N=5)** | **3 mg/kg (N=9)** | **10 mg/kg (N=10)** | **Total (N=28)** |
| --- | --- | --- | --- | --- | --- | --- |
| **Confirmed** |  |  |  |  |  |  |
| BOR |  |  |  |  |  |  |
| CR | 0 | 0 | 0 | 0 | 0 | 0 |
| PR | 0 | 0 | 0 | 0 | 0 | 0 |
| SD | 0 | 0 | 3 (60.0) | 7 (77.8) | 4 (40.0) | 14 (50.0) |
| PD | 1 (100.0) | 3 (100.0) | 2 (40.0) | 2 (22.2) | 3 (30.0) | 11 (39.3) |
| NE | 0 | 0 | 0 | 0 | 3 (30.0) | 3 (10.7) |
|  | | | | | | |
| ORR (CR + PR) | 0 (0.0) | 0 (0.0) | 0 (0.0) | 0 (0.0) | 0 (0.0) | 0 (0.0) |
| 95% CI | 0.00, 97.50 | 0.00, 70.76 | 0.00, 52.18 | 0.00, 33.63 | 0.00, 30.85 | 0.00, 12.34 |
|  | | | | | | |
| DCR (CR + PR + SD) | 0 (0.0) | 0 (0.0) | 3 (60.0) | 7 (77.8) | 4 (40.0) | 14 (50.0) |
| 95% CI | 0.00, 97.50 | 0.00, 70.76 | 14.66, 94.73 | 39.99, 97.19 | 12.16, 73.76 | 30.65, 69.35 |
| **Unconfirmed** | | | | | | |
| BOR |  |  |  |  |  |  |
| CR | 0 | 0 | 0 | 0 | 0 | 0 |
| PR | 0 | 0 | 0 | 1 (11.1) | 0 | 1 (3.6) |
| SD | 0 | 0 | 3 (60.0) | 6 (66.7) | 4 (40.0) | 13 (46.4) |
| PD | 1 (100.0) | 3 (100.0) | 2 (40.0) | 2 (22.2) | 3 (30.0) | 11 (39.3) |
| NE | 0 | 0 | 0 | 0 | 3 (30.0) | 3 (10.7) |
|  | | | | | | |
| ORR (CR + PR) | 0 (0.0) | 0 (0.0) | 0 (0.0) | 1 (11.1) | 0 (0.0) | 1 (3.6) |
| 95% CI | 0.00, 97.50 | 0.00, 70.76 | 0.00, 52.18 | 0.28, 48.25 | 0.00, 30.85 | 0.09, 18.35 |
|  | | | | | | |
| DCR (CR + PR + SD) | 0 (0.0) | 0 (0.0) | 3 (60.0) | 7 (77.8) | 4 (40.0) | 14 (50.0) |
| 95% CI | 0.00, 97.50 | 0.00, 70.76 | 14.66, 94.73 | 39.99, 97.19 | 12.16, 73.76 | 30.65, 69.35 |

*BOR* best overall response, *CI* confidence interval, *CR* complete response, *DCR* disease control rate, *NE* not evaluable, *ORR* overall response rate, *OS* overall survival, *PR* partial response, *PD* progressive disease, *PFS* progression-free survival, *SD* stable disease
